# Attributes of primary health care in the view of health professionals: a scoping review

**DOI:** 10.1590/1980-220X-REEUSP-2024-0149en

**Published:** 2024-12-06

**Authors:** Brenda Lorrana de Almeida Gomes, Renata Sant’ana Braga de Sousa, Renan Felipe Neves Mota, Cynthia Assis de Barros Nunes, Nayara Figueiredo Vieira, Nunila Ferreira de Oliveira, Roxana Isabel Cardozo Gonzalez, Valéria Pagotto

**Affiliations:** 1 Universidade Federal de Goiás, Faculdade de Enfermagem, Goiânia, GO, Brazil.; 2 Universidade Federal de Goiás, Faculdade de Enfermagem, Programa de Pós-graduação em Enfermagem, Goiânia, GO, Brazil.; 3 Universidade Federal de Catalão, Catalão, GO, Brazil.

**Keywords:** Primary Health Care, Nursing, Health Services Accessibility, Integrality in Health, Health Services Research, Atención Primaria de Salud, Enfermería, Accesibilidad a los Servicios de Salud, Integralidad en Salud, Investigación sobre Servicios de Salud

## Abstract

**Objective:**

To identify the scientific evidence on the performance of PHC, based on the presence and extent of its attributes, in the view of nurses and physicians.

**Method:**

Scoping review according to the recommendations of the Joanna Brigss Institute, carried out between January 2023 on eight databases, and updated in December 2023. The keywords “primary health care assessment” and “quality of primary health care” were used. Articles in any language were included, which used the PCATool-Brazil instrument, professional version, with a population of nurses and physicians.

**Results:**

Nineteen studies were included, published between 2012 and 2022, in nine Brazilian states. The overall average of PHC performance ranged from 6.5 to 8.2 and only two of the total indicated low performance of PHC services. Regarding the attributes, accessibility showed the worst performance and family orientation the best in all the studies. Specialization and statutory employment were predominantly frequent as associated factors.

**Conclusion:**

The results show that access to health services still needs to be strengthened and/or expanded and that, on the other hand, factors such as training and worker attachment strengthen PHC performance.

## RESUMEN

**Objetivo:**

Identificar las evidencias científicas sobre el desempeño de la APS, basado en la presencia y extensión de sus atributos, según la visión de los enfermeros y médicos.

**Método:**

Es una revisión de alcance conforme recomendaciones del Instituto Joanna Brigss, desarrollada entre enero de 2023 en ocho bases de datos y actualizada en diciembre de 2023. Las palabras clave utilizadas fueron *“primary health care assessment*” y “*quality of primary health care.* Se incluyeron artículos en cualquier idioma que utilizara el instrumento PCATool-Brasil, versión profesional, con población de enfermeros y médicos.

**Resultados:**

Se incluyeron 19 estudios publicados entre 2012 y 2022 en nueve estados brasileños. El promedio general del desempeño de la APS osciló entre 6,5 y 8,2 y del total; solo dos de los servicios de APS indicaron un valor bajo. En cuanto a los atributos, la accesibilidad tuvo el peor desempeño y la orientación familiar el mejor en todos los estudios. La especialización y el contrato estatutario fueron los factores asociados más frecuentes.

**Conclusión:**

Los resultados demuestran que el acceso a la salud necesita fortalecerse y/o ampliarse y que factores como la capacitación y el vínculo laboral fortalecen el desempeño de la APS.

## INTRODUCTION

Primary Health Care (PHC) is considered the gateway to health services for the Brazilian population^(1)^ and the organizer of the Health Care Network (HCN)^(2)^. Its consolidation and implementation as a nationwide policy in Brazil represents one of the most significant advances of the Unified Health System (SUS) in recent decades^(1,2)^.

Due to its magnitude and roles in the HCN, PHC has grown from 8,000 teams in 2007 to more than 42,000 family health teams in 2017^(3)^, leading to the need to evaluate its performance in different dimensions. In addition, given the epidemiological and demographic changes in the population, as well as in work processes and the supply of services in PHC over time, evaluation becomes imperative in order to monitor the effectiveness of its actions^(3,4)^. Evaluative research in Brazil has boosted knowledge about PHC and shows that strong PHC leads to greater user satisfaction and, consequently, improved health indicators^(4)^.

One of the proposals for assessing the performance of PHC services is through its structuring elements, defined as attributes, which are classified as essential (first contact access, longitudinality, comprehensiveness and coordination of care) and derived (community and family orientation)^(5)^. The Primary Care Assessment Tool (PCATool) is the instrument that measures the presence and extent of PHC attributes, from the point of view of professionals (nurses and physicians) and users^(6)^. The PCATool is based on a quality assessment model based on aspects of the structure, process and outcome of the services assessed^(7)^.

In Brazil, the applicability of the PCATool for evaluating PHC performance has been carried out in different parts of the country at municipal, regional and micro-regional level, with both users and professionals^(6)^. In 2017, a systematic review brought together 22 studies with users and showed that longitudinality was the attribute with the worst performance^(8)^.

From the point of view of professionals, some studies have also been carried out using the PCATool^(9, 10, 11, 12)^, but no review studies have been found which bring together the evidence on PHC performance from the point of view of health professionals. This study therefore makes it possible to identify the available evidence related to the evaluation of PHC services, based on the presence and extent of their attributes, broadening the recognition of weaknesses and potentialities. The aim of this study was to identify scientific evidence on the performance of PHC, based on the presence and extent of its attributes, in the view of nurses and physicians.

## METHOD

### Study Design

This is a scoping review, based on the methodology proposed by the Joanna Briggs Institute^(13)^. The study was developed through five stages: definition of the research question; screening of relevant studies; selection of studies, data extraction; analysis, presentation and synthesis of the results. The recommendations set out in the Prisma Extension for Scoping Reviews (Primas-ScR)^(14)^ were also used.

### Guiding Question

The mnemonic strategy PCC (Population, Concept and Context) was used to develop the guiding question. For this study, we considered PHC nurses and physicians as the population (P); the concept of interest (C) was the evaluation of the presence and extent of PHC attributes using the PCATool-Brazil; and the context (C) was PHC. Thus, the central question of this scoping review was: “What is the performance of PHC services in Brazil, based on the presence and extent of their attributes, in the view of nurses and physicians?

### Selection Criteria

This review considered analytical observational studies (cross-sectional and cohort) that used the PCATool-Brazil instrument, professional version. Included were articles published in any language, not limited in time, with the descriptor in any field. After identifying the studies in the databases, the following were excluded: duplicate studies, protocols, research projects or previews and studies whose objective did not correspond to the research question.

### Data Collection

A bibliographic selection was carried out using the databases Scopus, Online Medical Literature Search and Analysis System (MEDLINE) (accessed via PubMed), Embase, CINAHL, BDENF, LILACS and SCIELO. The articles were collected in January 2023. The following keywords were used: “primary health care assessment” OR “quality of primary health care”.

All the articles found in each of the databases were downloaded in Excel spreadsheet format and grouped in a single spreadsheet. Duplicate articles were excluded using Excel’s duplicate removal tool from the article title column. The titles, abstracts and full texts were read independently by two reviewers and, in the event of disagreements, the publications were analyzed by a third researcher. The eligible articles were read in full and those that met the inclusion criteria were included.

### Data Analysis

Data was extracted using a form developed by the authors and based on the form suggested by the JBI manual^(13)^. The data extracted included title, authors, year of publication, country in which the study was carried out, type of study, study objectives, study methods, population and sample size, scope of the study (municipal, state or national), general results (including overall PCATool-Brazil score, attribute with lowest and highest score, and associated factors investigated through regression analysis).

Based on the data extracted, a descriptive analysis of the information collected was carried out using frequency distribution. Two tables were also drawn up: one with the data from the selected publications and the other summarizing all the values found for the PHC attribute scores.

## RESULTS

**Figure 1:**
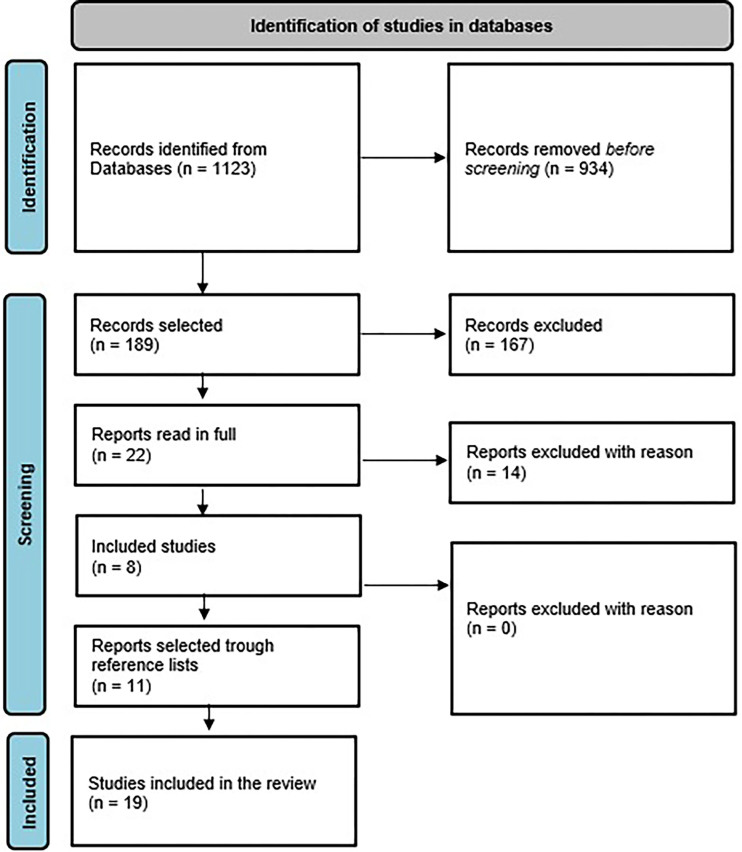
Flowchart of the search for articles, according to PRISMA-ScR recommendations, on the process of selecting publications for the review, Brazil, 2023.

Of the 1123 articles identified, 189 were selected for abstract reading. Of these, 4 were excluded due to duplication and 163 that did not cover the subject of the study. Twenty-two articles were eligible for reading, of which 14 were excluded for the following reasons: including managers in the calculation of the PCATool score and using users as the population. The list of references of these articles was analyzed and 11 articles were included in this review. In the end, 19 articles were included (Figure 1).

Chart 1 summarizes the articles included in the review, showing where the studies were carried out, the objectives, the population, the scope of the studies and the main results.

**Chart 1:** Summary of the articles included in the review – Goiânia, GO, Brazil, 2023.

	Author	Country/State/City	Objective	Population	Scope of the study	Results
**1**	Costa et al.^(15)^	Brazil Minas Gerais Juiz de Fora	Evaluate the application of PHC attributes from the perspective of medical professionals at primary healthcare units	94 physicians	Municipal 63 primary care centers (49 urban and 14 rural)	**Overall score:** 7.05. **Highest score:** family orientation (8.72 SD 1.76) and coordination/information system (8.72 SD 1.96). Lowest score: accessibility (4.02 SD 3.99). **Associated factors:** not analyzed. **Positive correlation between:** (i) higher scores in the longitudinality and coordination/integration of care attributes and longer time working in PHC and; (ii) higher scores in the longitudinality and family orientation attributes for those who attended postgraduate courses (specialization, residency or master’s).
**2**	Bomfim et al.^(16)^	Brazil Mato Grosso do Sul 29 municipalities	Analyzing the quality of adult health care in PHC services from the perspective of users and professionals	176 nurses 56 physicians	State 29 municipalities represented by 4 macro-regions of the state of Mato Grosso do Sul	**Overall score:** 7.11 (SD 1.05) **Highest score:** Family orientation 8.57 (SD 1.96) **Lowest score:** Accessibility 3.94 (SD 1.31) **Associated factors:** not analyzed. When comparing the scores of professionals with those of users, there were significant differences in all attributes.
**3**	Maia et al.^(12)^	Brazil Goiás 10 municipalities	To evaluate PHC based on its attributes, from the perspective of professionals, verifying factors that are associated with better care.	41 nurses 31 physicians	Regional 10 municipalities in the state of Goiás. 36 PHC and 45 family health teams from the 10 municipalities.	**Overall score:** 7.26 (SD 0.78) **Highest score:** Family orientation (8.82 SD 1.3) **Lowest score:** Accessibility 3.71 (SD 1.26) **Associated factors:** Being a doctor in the More Doctors Program (MDP) was associated with the general (β = 0.942; p = 0.001), essential (β = 0.948; p = 0.001) and derived (β = 1.066; p = 0.006) scores.
**4**	Costa et al.^(17)^	Brazil Maranhão São José de Ribamar	Evaluate the quality of PHC services in the municipality.	73 nurses and physicians	Municipal 35 PHC 44 teams	**Overall score:** 7.54 (SD not shown) **Highest score:** coordination/information system (8.93 SD 0.4). **Lowest score:** accessibility (3.91 SD 0.4). **Associated factors:** not analyzed. When comparing the scores of professionals with those of users, there were significant differences in all attributes.
**5**	Silva and Alves^(18)^	Brazil Minas Gerais Diamantina	To evaluate the degree of implementation of as an indicator of the quality of care provided to children. children.	8 nurses 14 physicians	Municipal 14 teams (8 urban, 4 rural and 2 mixed)	**Overall score:** 6.9 (SD 1.1) **Highest score:** comprehensiveness, information systems (9.0) and family orientation (8.9). **Lowest score:** accessibility (4.2). **Associated factors:** not analyzed. A comparison test between the performance of services in urban and rural areas showed that accessibility had a higher score among professionals in rural areas (5.3) than those in urban areas (3.7) (p = 0.042).
**6**	Vieira et al.^(19)^	Brazil Minas Gerais Lagoa Santa	Compare the presence and extent of PHC attributes in the overall and performance of HCAs.	17 nurses 11 physicians	Municipal Lagoa Santa 17 family health teams	**Overall score:** 6.8 (SD 0.9) **Highest score:** family orientation (8.6 SD 1.3) **Lowest score:** accessibility (3.8 SD 1.0). **Associated factors:** not analyzed.
**7**	Gomes and Fracolli^(20)^	Brazil São Paulo Municipalities in the Presidente Prudente region	To assess whether the essential and derived attributes of Primary Health Care are present in the Family Health Strategy.	44 nurses 39 physicians	Regional 21 municipalities of the Regional Health Care Network of Presidente Prudente	**Overall score:** 7.88 (SD 0.90) **Highest score:** Coordination-information system 8.74 (1.42) **Lowest score: Accessibility:** 5.57 (1.42) **Associated factors:** not analyzed. Shows only the scores for the attributes accessibility, longitudinality and coordination-integration of care.
**8**	Piovesan et al.^(21)^	Brazil Rio Grande do Sul	To evaluate, in the experience of professionals, the quality of primary health care in municipalities where children and adolescents with HIV come from and are followed up in specialized services.	167 nurses 245 physicians	State 25 municipalities in RS	**Overall score:** 6.64 (SD 1.02) **Highest score:** coordination, information system (8.19 (1.87 in PHC and 8.24 (1.50) in FHS). **Lowest score:** first contact access (3.89 SD 11.78 in PHC and 3.80 (1.09) in FHS). **Associated factors:** Female gender (p = 0.009); general practitioner training (p < 0.001); employment in the statutory service (0.029); the professional’s job position as service coordinator (p = 0.087) were associated with better PHC performance.
**9**	Santos et al.^(11)^	Brazil Municipality in the southwest of Goiás	To assess the presence and extent of PHC attributes by health professionals working in PHC in a municipality in southwestern Goiás.	17 nurses 13 physicians	Municipal 16 primary care centers in a municipality in southwestern Goias	**Overall score:** 7.2 (no PD) **Highest score:** Family orientation 8.7 **Lowest score:** Accessibility: 3.4 **Associated factors:** time in practice ≤ 5 years and time in practice ≥ 6 years; health professionals with and without specialization in the area of practice
**10**	Sousa et al.^(22)^	Brazil Pará Canaã dos Carajás	To evaluate the presence and extent of the essential and derived attributes of primary health care in the leprosy control program, from the perspective of nurses.	11 nurses	Municipal 8 FHS teams 3 PHC 6 FHS	**Overall score:** 8.2 (±0.7) **Highest score:** Comprehensiveness - services available 9.5 (±0.4) **Lowest score:** Access 5.9 (±1.4) **Associated factors:** not analyzed. Descriptive analysis only.
**11**	Oliveira et al.^(23)^	Brazil Goiás Northwest region of Goiânia	Analyze the training and qualification profile of Family Health Strategy (FHS) professionals and the factors associated with the quality of PHC services in the northwestern region of Goiânia.	44 nurses 48 physicians	Municipal 18 primary care centers in the northwest region of Goiania	**Overall score:** 6.7 (1.0) **Highest score:** Comprehensiveness - services provided: 8.0 (1.4) **Lowest score: Accessibility:** 4.6 (1.0) **Associated factors:** Analyzed, but after adjusted analysis, specialization, training and professional development were not associated with a high score.
**12**	Ferreira et al.^(10)^	Brazil Minas Gerais Passos	To evaluate, using the PCATool instrument, the extent of the essential and derived attributes of PHC from the perspective of nurses from FHSs and conventional PHCs in the municipality of Passos, MG.	27 nurses	Municipal 19 FHS 9 PHC	**Overall score:** For PHC 6.8 (1.16), for FHS 7.4 (0.64) **Highest score:** Family orientation PHC 8.61 (1.42), FHS 9.18 (1.16) **Lowest score: Accessibility:** PHC 4.44 (0.97), FHS 4.21 (0.86) **Associated factors:** not analyzed. Descriptive analysis only.
**13**	Nascimento et al.^(24)^	Brazil Rio Grande do Sul Santa Maria	To evaluate the quality of health care for children and adolescents living with HIV among the different types of PHC service in Santa Maria (RS) using the PCATool-Brazil, Professional version.	34 nurses 60 physicians 24 dental surgeons	Municipal 31 PHC services 18 PHC 13 FHS	**Overall score:** 6.49 (1.15) **Highest score:** coordination, information system 8.44 (1.61) **Lowest score: access** 4.02 (1.30) **Associated factors:** The following were associated with a high score (>6.6): having another job, working in the FHS and having a statutory contract.
**14**	Turci et al.^(25)^	Brazil Minas Gerais Belo Horizonte	To evaluate PHC performance in Belo Horizonte, Minas Gerais, Brazil, using the PCATool questionnaire among family health team nurses and managers.	463 nurses *138 managers	Municipal 538 teams 147 PHC	**Overall score:** 0.75 (CI 0.74-0.76). **Highest score:** Access, first contact (0.95 CI 0.94-0.97) **Lowest score:** accessibility (0.45 CI 0.43-0.46) **Associated factors:** Availability of equipment and other supplies (adjusted PR = 1.57). Training of professionals in family health (PR = 1.44). Physicians working more than 30 hours a week (PR = 1.42). 4 or more teams per basic health unit (PR = 1.09).
**15**	Silva et al.^(26)^	Brazil Minas Gerais Health micro-region Alfenas	To evaluate the attributes of PHC by triangulating and comparing the points of view of the social actors involved in the care process.	19 nurses 15 physicians	Regional 11 municipalities in a micro-region in the state of Minas Gerais 33 FHS centers	**Overall score:** 7.40 (SD 0.77) **Highest score:** Coordination, Information systems (8.95 SD 1.33) **Lowest score**: First Contact Access, Accessibility (4.13 SD 1.27) **Associated factors:** not analyzed. The attributes were compared between the adult groups, caregivers of children aged 0 to 2 and professionals, showing agreement in the low assessment for First Contact Access, Accessibility and high for Longitudinality.
**16**	Araújo et al.^(27)^	Brazil Federal District	To evaluate the presence and extent of attributes in comprehensive child health care, comparing PCCTs that work with the traditional model with PCCs in the Federal District, in the perception of users and professionals.	16 professionals (eight per PHC model)	Regional Two regions of the Federal District 15 PCCT 19 FHS	**Overall score:** PCCT 7.01 (0.54); FHS 6.62 (0.61) **Highest score:** PCCT coordination 9.19 (0.88); FHS family orientation 8.55 (1.64) **Lowest score:** Accessibility: PCCT 3.21 (0.96); FHS 2.75 (0.65) **Associated factors:** not analyzed.
**17**	Chomatas et al.^(28)^	Brazil Paraná Curitiba	To evaluate the presence and extent of the attributes of primary health care in the municipality of Curitiba, comparing traditional centers with Family Health Strategy units, through the experience of their health professionals.	99 nurses 91 physicians	Municipal 45 units with FHS 47 PCCT	**Overall score:** PCCT 6.7 (6.6–6.8); FHS 7.4 (7.3–7.5) **Highest score:** PCCT Coordination 8.3 (8.1–8.5); FHS Coordination 8.5 (8.2–8.7) and Family Orientation 8.5 (8.3–8.7) **Lowest score:** PCCT Accessibility 4.1 (3.9–4.2); FHS Accessibility 4.2 (4.0–4.4) **Associated factors:** units with FHS (PR = 1.44) were associated with a high overall PHC score. Being a medical professional compared to a nurse (PR = 0.87) was associated with a lower prevalence of high overall PHC score. In the stratified multivariable models, among physicians, the presence of a high general PHC score was positively associated with a unit with an FHS and the number of weekly consultations (10 weekly consultations). For nurses, having training in community nursing was positively associated with the presence of a high overall PHC score
**18**	Vitória et al.^(29)^	Brazil Santa Catarina Chapecó	To assess the suitability of the elements of the structure and processes of PHC in Chapecó.	47 nurses 51 physicians	Municipal 24 PHC	**Overall score:** 7.09 (6.95–7.24) **Highest score:** Coordination - information system: 8.8 (8.55–9.07) **Lowest score:** Access 3.6 (3.40–3.90) **Associated factors:** Not analyzed. Discusses training and work process, but does not relate them to the scores.
**19**	Castro et al.^(9)^	Brazil Rio Grande do Sul Porto Alegre	Comparing the quality of adult health care between the different types of PHC services in Porto Alegre, Rio Grande do Sul, Brazil Alegre, Rio Grande do Sul,	114 nurses 226 physicians	Municipal 26 PHC 31 FHS	**Overall score:** PHC 6.58, FHS 7.08 **Highest score:** In both IS Coordination 9.00 and 9.02 Lowest score: Both Access 3.14 and 3.53 **Associated factors:** Specialty in the PHC area and continuing education offered by the service were associated with a high overall score.

The period of publication ranged from 2012 to 2022, with 65% in the last 6 years. As for the locations, the studies were carried out in 9 Brazilian states, with the highest concentration in Minas Gerais (6 studies), followed by Goiás (3 studies), Rio Grande do Sul (3 studies), Maranhão, Mato Grosso do Sul, Pará, São Paulo, Paraná, Santa Catarina and the Federal District (1 study each). In 68.4% of the studies, the scope was municipal and in the others it was regional and/or statewide (Chart 1).

As for the population, one study included only physicians, three only nurses, 11 nurses and physicians, and four nurses, physicians and dental surgeons. In addition, two studies compared the views of professionals with those of users^(16,17)^, and one study compared users with caregivers^(18,26)^. Two studies also compared the views of managers with those of professionals^(20,25)^. These studies were included because they presented the PCATool score from the point of view of professionals, separate from the other groups (users, caregivers and managers), which were not considered in the analysis of this review (Chart 1).

It is noteworthy that in describing the objectives, in eight studies the authors use the terms evaluation of the presence and extent of PHC attributes, in six evaluation of the quality of PHC services, one mentions PHC performance, one the degree of implementation of the attributes, and in the others other descriptions similar to the first two (Chart 1).

With regard to the associated factors presented, statistical analyses were carried out in six studies, allowing independent variables to be inferred to be associated with the PCATool score^(9,11,25,28)^. The variable having specialization in the area of work was associated with higher performance in PHC services in four studies^(9,11,25,28)^. Having a statutory employment contract was associated with a higher PHC score in two studies^(21,24)^. Working in primary care centers with a family health strategy was associated in two studies^(25,28)^ (Chart 1).

Some variables related to the medical professional, such as being a doctor in the More Doctors Program (MDP)^(12)^; general practitioner training^(21)^; presence of the physician for more than 30 hours a week^(25)^ were associated. Other variables were associated, but each in one study: female gender^(25)^, being the coordinator of the service^(21)^, time working ≤ 5 years^(11)^, having another job^(24)^, availability of equipment and other supplies^(25)^, being a medical professional in relation to a nurse^(28)^, provision of continuing education by the service^(9)^. Table 1 shows the scores of the attributes, with heterogeneity in the presentation of the values. All the studies presented the overall PCATool Brazil score, which ranged from 6.49 to 8.20. Of the 19 studies, only two had a score below the instrument’s cut-off point (6.6)^(9,24)^.

**Table 1:** Primary Health Care attributes score – Goiânia, GO, Brazil, 2023.

	Studies	Essentials attributes mean (SD)				Derived attributes mean (SD)		Overall score mean (SD)		
		Accessibility	Lengitudinality	Coordination	Comprehensiveness	Family orientation	Community orientation	Essential	Derivatives	General
**1**	Costa et al.^(15)^	4.02 (3.99)	7.12 (2.62)	7.22 (3.08)^CI^ 8.72 (1.96)^SI^	6.74 (3.77)^SD^ 8.31 (4.25)^SP^	8.72 (1.76)	7.57 (3.00)	6.93 (3.47)	7.95 (2.70)	7.05 (3.40)
**2**	Bomfim et al.^(16)^	3.94 (1.31)	7.7 (1.42)	7.49 (1.78)^CI^ 8.46 (1.71)^SI^	7.1 (1.49)^SD^ 6.63 (2.26)^SP^	8.57 (1.96)	7.03 (2.09)	–	–	7.11 (1.05)
**3**	Maia et al.^(12)^	3.71 (1.26)	7.29 (1.29)	7.24 (1.48)^CI^ 8.48 (1.45)	7.32 (1.02) 8.04 (1.35)	8.82 (1.31)	7.19 (1.69)	7.01 (0.75)	8.00 (1.16)	7.26 (0.78)
**4**	Costa et al.^(17)^	3.91 (0.4)	7.57 (*)	8.15 (*)^CI^ 8.93 (0.4)	7.51 (*) 8.19 (*)	8.90 (0.4)	7.58 (*)	7.28 (0.2)	8.02 (0.3)	7.54 (*)
**5**	Silva and Alves^(18)^ urban	3.7 (0.3)**	7.1 (0.5)	6.7 (0.5)^CI^ 8.8 (0.4)^SI^	5.9 (0.6)^SD^ 7.6 (0.5)^SP^	9.2 (0.3)	5.8 (0.4)	6.6 (0.4)	–	6.9 (0.3)
	Silva and Alves^(18)^ rural	5.3 (0.6)**	5.6 (0.7)	6.2 (0.4)^CI^ 9.3 (0.4)^SI^	5.6 (0.6)^SD^ 7.0 (0.6)^SP^	8.0 (0.8)	5.7 (0.8)	6.5 (0.4)	–	6.6 (0.4)
	Silva and Alves^(18)^ General	4.2 (1.4)	6.59 (1.76)	8.99 (1.18) 6.03 (1.87)	6.03 (1.87) 7.46 (1.68)	8.94 (1.26)	5.79 (1.74)	6.7 (1.2)	6.86 (1.10)	6.9 (1.1)
**6**	Vieira et al.^(19)^	3.8 (1.0)^A^	6.9 (1.1)	7.8 (1.0)^CI^	7.6 (1.1)^SD^ 6.6 (1.6)^SP^	8.6 (1.6)	6.5 (1.4)	6.5 (0.8)	7.6 (1.2)	6.8 (0.9)
**7**	Gomes and Fracolli^(20)^	5.57 (1.42)	7.71 (1.26)	7.70 (1.23)^CI^ 8.74 (1.42)^SI^	8.29 (1.24)^SD^ 8.40 (1.57)^SP^	8.53 (1.74)	8.12 (1.41)	7.73 (0.85)	–	7.88 (0.90)
**8**	Piovesan et al.^(21)^ PHC	3.96 (1.78)	6.74 (1.34)	7.03 (1.51)^CI^ 8.19 (1.87)^SI^	6.49 (1.77)^SD^ 6.37 (2.77)^SP^	–	–	6.47 (1.10)	–	–
	Piovesan et al.^(21)^ FHS	3.80 (1.09)	7.17 (1.24)	6.87 (1.50)^CI^ 8.24 (1.50)^SI^	7.17 (1.52)^SD^ 7.66 (1.96)^SP^	–	–	6.82 (0.88)	–	–
**9**	Santos et al.^(11)^ ***	3.4	7.0	7.1^CI^ 8.4^SI^	7.3^SD^ 8.2^SP^	8.7	7.2	–	–	7.2
**10**	Sousa et al.^(22)^	5.9 (1.4)	8.1 (1.6)	7.8 (1.9)	9.5 (0.4)^SD^ 8.8 (1.0)^SP^	7.8 (2.8)	7.8 (1.8)	8.1 (0.6)	7.9 (1.6)	8.2 (0.7)
**11**	Oliveira et al.^(23)^	4.6 (1.0)	6.8 (1.2)	6.9 (1.3)^CI^ 6.5 (1.4)^SI^	6.5 (1.4)^SD^ 8.0 (1.4)^SP^	7.6 (1.6)	6.5 (1.4)	6.5 (0.9)	6.9 (1.4)	6.7 (1.0)
**12**	Ferreira et al.^(10)^ PHC	4.44 (0.97)	7.37 (1.20)	7.57 (0.84)^CI^ 7.36 (2.65)^SI^	5.42 (0.96)^SD^ 7.69 (1.69)^SP^	8.61 (1.42)	5.83 (2.94)	6.65 (1.06)	–	6.80 (1.16)
	Ferreira et al.^(10)^ FHS	4.21 (0.86)	7.88 (1.17)	7.66 (1.18)^CI^ 8.71 (1.82)^SI^	6.13 (0.54)^SD^ 7.92 (1.18)^SP^	9.18 (1.16)	7.43 (1.34)	7.09 (0.68)	–	7.40 (0.64)
**13**	Nascimento et al.^(24)^	4.02 (1.30)	6.70 (1.37)	6.13 (1.54)^CI^ 6.45 (3.04)	6.97 (1.56) 8.44 (1.61)	7.80 (2.36)	5.41 (2.03)	6.45 (1.06)	6.61 (1.88)	6.49 (1.15)
**14**	Turci et al.^(25)^ ^¥^	0.45 (0.43–0.46)	0.83 (0.81–0.85)	0.78 (0.77–0.79)	0.83 (0.82–0.84)	0.68 (0.66–0.71)	0.56 (0.54–0.59)	–	–	0.75 (0.74–0.76)
**15**	Silva et al.^(26)^	4.13 (1.27)	7.86 (1.12)	6.91 (1.47) 8.95 (1.33)	6.80 (1.24) 8.01 (1.25)	8.82 (1.40)	7.68 (1.46)	5.92 (1.20)	–	7.40 (0.77)
**16**	Araújo et al.^(27)^ PCCT	3.21 (0.96)	7.53 (1.24)	7.61 (1.60)^CI^ 9.19 (0.88)^SI^	5.97 (0.97)^SD^ 8.36 (1.70)^SP^	7.57 (2.10)	6.67 (2.39)	6.98 (0.64)	7.11 (0.21)	7.01 (0.54)
	Araújo et al.^(27)^ FHS	2.75 (0.65)	7.31 (1.42)	6.69 (1.83)^CI^ 8.37 (1.36)^SI^	5.31 (1.10)^SD^ 8.35 (1.53)^SP^	8.55 (1.64)	5.61 (1.57)	6.46 (0.65)	7.08 (0.64)	6.62 (0.61)
**17**	Chomatas et al.^(28)^ PCCT	4.1 (3.9–4.2)	6.0 (5.8–6.1)	6.9 (6.7–7.0)^CI^ 8.3 (8.1–8.5)^SI^	7.1 (7.0–7.2)^SD^ 6.0 (5.7–6.2)^SP^	7.8 (7.6–8.0)	7.6 (7.4–7.8)	6.4 (6.3–6.5)	7.7 (7.5–7.9)	6.7 (6.6–6.8)
	Chomatas et al.^(28)^ FHS	4.2 (4.0–4.4)	6.6 (6.4–6.7)	7.0 (6.8–7.1)^CI^ 8.5 (8.2–8.7)^SI^	7.9 (7.8–8.0)^SD^ 8.3 (8.2–8.5)^SP^	8.5 (8.3–8.7)	8.1 (7.9–8.3)	7.1 (7.0–7.2)	8.3 (8.2–8.5)	7.4 (7.3–7.5)
**18**	Vitória et al.^(29)^	3.6 (3.40–3.90)	6.0 (5.83–6.34)	7.2 (6.97–7.53)^CI^ 8.8 (8.55–9.07)^SI^	7.7 (7.57–7.96)^SD^ 7.6 (7.33–7.89)^SP^	8.6 (8.32–8.91)	6.9 (6.65–7.26)	6.8 (6.71–7.01)	–	7.09 (6.95–7.24)
**19**	Castro et al.^(9)^ PHC	3.14	6.21	6.92^CI^ 9.00^SI^	6.29^SD^ 7.02^SP^	8.21	5.58	6.45	–	6.58
	Castro et al.^(9)^ FHS	3.53	6.53	7.17^CI^ 9.02^SI^	6.50^SD^ 8.27^SP^	8.87	6.75	6.84	–	7.08

*Study by Costa et al.^(17)^: missing Standard Deviation (SD) values for these attributes;

^§^Study by Vieira et al.^(19)^: missing information due to missing attributes.

^¥^Study by Turci et al.^(25)^: scores by regression;

**Study by Silva and Alves^(18)^: Standard Error (SE) value;

***Study by Santos et al.^(11)^: no Standard Deviation (SD) values.

In relation to the essential attributes, 16 studies (84.2%) presented an overall essential score ranging from 5.9 to 8.1; while the overall score for the derived attributes was presented in 10 studies (52.6%), ranging from 4.3 to 8.3. It should be noted that the overall essential score was lower than the derived scores in all but two studies^(19,22)^.

Among the four essential attributes (accessibility, longitudinality, comprehensiveness and coordination of care), the lowest scores were observed for accessibility, whose values were below the cut-off point in all the studies. The longitudinality score ranged from 5.6 to 8.1, and in 6 studies (31.6%) it was below 6.6. The coordination of care and comprehensiveness attributes had higher scores than the first two, with values above 8.5 in 17 (89.4%) and 11 (58.0%) studies respectively. As for the two derived attributes (family and community orientation), in all the studies the scores were above 7.5 for family orientation. As for community orientation, in 8 studies (42.1%), the score was below 6.6. The attributes of coordination of care and comprehensiveness had higher scores than the first two. In the case of the coordination of care attribute, 11 (57.9) articles had scores above 8.5. As for the two derived attributes (family and community orientation), most of the studies showed scores above 7.5 for family orientation. As for community orientation, in 10 studies (52.6%), the score was below 6.6.

In relation to the studies that presented different analyses according to the type of health center, those that compared the scores of the attributes in the FHS and PCC showed that in the FHS the scores were higher in most of the attributes^(8,12,19)^. The study that carried out a separate analysis between rural and urban FHSs showed that the performance of urban FHSs was superior to rural ones^(5)^.

## DISCUSSION

The results of this scoping review show that the PCATool has been used as a tool to assess the presence and extent of PHC attributes in different national contexts. Although the overall score for the attributes is homogeneous between the different locations, the average values for each of the attributes are heterogeneous, indicating that the performance of PHC services in Brazil may be linked to the form of organization and involvement of management in the PHC work process, showing that despite policy recommendations there are specific aspects that need to be improved in each of the attributes according to the reality of each service.

Despite this, in the studies included, the largest proportion showed high performance in the essential and derived attributes (above 6.6). The Family Health Strategy is one of the structuring programs of the Unified Health System (SUS), and its expansion has contributed to changes in the epidemiological profile of the population over the last three decades^(30)^. Studies analyzing health indicators in this period, which corresponds to a large part of the existence of the SUS, show a drop in mortality from communicable diseases, maternal and child morbidity and mortality and an increase in the population’s healthy life expectancy^(31,32)^, as well as a lower risk of death in people who use PHC^(33)^. The results of this study, together with evidence from other studies showing significant changes in health indicators, reaffirm the role of PHC as the gateway to the SUS, as the coordinator of care and as the center of communication with the entire care network, indicating the sustainability and consolidation of this model of health care adopted in Brazil^(34,35)^.

Most of the studies in this review showed that professionals evaluated the essential attributes of PHC as strongly oriented. However, from the users’ perspective, a systematic review of 22 articles between 2007 and 2015 showed a better evaluation of the “first contact access” attribute, while a worse evaluation was given to the accessibility, comprehensiveness, family orientation and community orientation dimensions^(8)^. Professionals’ view of PHC attributes has a different perspective when compared to that of users, and although both are substantial for understanding the performance of PHC services, in this article, the focus will be on evaluating the perspective of health professionals.

The low performance of the accessibility attribute in the view of professionals was evidenced in all the studies included. Access can be differentiated into two broad dimensions, geographical and socio-organizational, while the first encompasses the factors that can increase the difficulty of reaching the health service, such as distance, time and cost of travel, the second refers to all the other characteristics that involve access, such as how to enter and receive care at the health unit^(7)^. In the PCATool, the nine questions that make up the second dimension include items such as opening hours on weekends, at night, counseling by virtual communication tool (such as messaging apps)^(6)^, which in addition to being different in the various Brazilian scenarios, may not be the reality of local health policy. Data from the third cycle of the Primary Care Quality Improvement Program (PMAQ), 2017-2018, in its organizational component showed that health units operate predominantly from Monday to Friday, with little available at alternative times (1.6% of them operated on Saturdays and 0.7% on Sundays)^(36)^. It is noteworthy that in the users’ evaluation, the accessibility component also showed low performance in a systematic review^(8)^, and the questions are similar to the professional version. In general, the difficulties that prevent access to health services can manifest themselves in subtle, obvious or hidden ways, which when identified do not receive effective referrals for their solution. This highlights the need to improve processes to guarantee people’s entry and quality care in PHC, given the recognized role of this attribute in reducing morbidity and mortality.

Among the essential attributes, coordination of care had the highest score among the studies analyzed. Coordination refers to the articulation between the various levels of care, which is established through lines of communication that allow the flow of information between professionals at the various levels of care. With this attribute developed, it is possible to continuously monitor the health of each patient^(5)^. As such, it reflects the capacity of the HCN to provide solutions and to function, as well as the structural capacity of the policies for a strong PHC. The satisfactory evaluation shown in this attribute can perhaps be attributed to the perception of the need to involve the primary and specialized healthcare network in the health of the local population. However, it should be pointed out that ordering from PHC does not always contribute as a barrier, but the return of the user to the PHC service, known as counter-referral, is an aspect that hinders continuity of care, and is not always integrated or communicated, which could be further explored in specific research on the coordination of care.

The family orientation attribute performed highly in all the studies included in this review. It involves professional-user-family interaction in making decisions about health care and treatment, identifying problems in the family, as well as assessing the professional’s willingness to address issues that are important to health^(6)^. Family orientation is effective when achieving comprehensiveness provides a basis for considering the individual within their environment; assessing needs for comprehensive care considers the family context and its exposure to health threats; the challenge of coordinating care is faced with limited family resources, requiring actions to be tailored to the family context. From this perspective, the FHS’s assumptions of reorganizing health care practice through a family-centered care model with longitudinal follow-up are once again highlighted. The high performance pointed out in all the studies by the health professionals may be a reflection of the professional training that is oriented towards this model of care, with an emphasis on a broader approach to the person and their family.

However, it is also important to assess the users’ perspective on family orientation, since for a strong and resolutive PHC, it is important that both professionals and users and families are satisfied and engaged with the care, which, according to research on this subject with users^(18,37)^ still needs to be better worked on and improved. It is also important to highlight the change in the inclusion of different attributions for PHC professionals provided for in the PNAB 2017, which, by decentralizing actions provided for at other points in the HCN, can overload services and consequently lead to individualized care to the detriment of that centered on the person and their family. Thus, as previous research has pointed out, it is important for professionals to get closer to the family and social context and to include the family in the planning of interventions, as well as the relevance of this topic for training and the promotion of permanent health education^(18)^.

Among the variables that were evaluated as associated factors in the 19 studies, having a specialization and having a statutory contract were the most frequent, and will be discussed for their potential impact on PHC.

Specialization was found in four studies^(9,11,25,28)^. A study of 20 professionals who had specialized in education for the SUS indicated that the training qualified the work process and added greater dynamism to meet collective and individual demands, as well as bringing about changes in the management profile, highlighting teamwork and interdisciplinary proposals^(38)^. The direction of generalist training proposed by the DCN, with a view to strengthening work within the SUS, can take a dichotomous approach between generalist skills and specialties, to the detriment of the possibility of directing career training towards PHC, which encompasses the needs and complexities inherent in this work context^(39)^. Generalist training does not have a direct impact on whether medical professionals choose to follow this career path or remain in primary care. This path tends to be temporary and contributes to training aimed at developing social aspects and contributing to society; however, these professionals tend to seek specialized training, usually because of the justification of greater professional recognition^(40)^. These results show the importance of generalist, quality training that is closer to the reality of health services and to strengthening the SUS, but they also show the need to invest in specific training throughout their careers, including specialization(s) focused on PHC in order to improve team performance.

The variable of statutory employment was found in two studies^(21,24)^, a positive result for PHC, since professional stability makes the attribute of longitudinality effective and promotes the strengthening of the bond between the professional and the user^(41)^. However, it is worth mentioning that there is evidence that administrative contracts are also associated with high PHC performance^(42)^. It should therefore be pointed out that professional turnover is an obstacle to the expansion and consolidation of PHC.

### Limitations of the Study

One limitation of the study refers to the procedures for assessing the evidence found. However, all the studies were cross-sectional, an appropriate design for using the PCATool instrument, the focus of this review. In addition, it is possible that some relevant studies were not covered by the limitations of the keywords, however we believe that any studies missed do not substantially alter the pattern of findings.

## CONCLUSION

This study made it possible to synthesize knowledge about the presence and extent of PHC attributes from the point of view of health professionals, nurses and physicians. The results are important for recognizing the scenario of essential and derived attributes in the different regions of the country, which can help nurses and physicians who work in the care and management of PHC services to reflect on the reorganization of the work process, based on its structuring elements, which are the attributes.

Thus, attributes such as first-contact access, which performed poorly in most of the studies, can be a starting point for identifying key problems for reorganizing actions in PHC services, which will drive the development of actions that promote longitudinality, comprehensiveness and coordination of care. It is suggested that primary studies on the quality of care in PHC use other evaluation methodologies, both quantitative and qualitative. In this way, phenomena that were not covered by the PCATool instrument could be identified.
