# Supplementary material for: Attributes of primary health care in the view of health professionals: a scoping review
Source: Rev Esc Enferm USP. 2024 Dec 6;58:e20240149. doi: 10.1590/1980-220X-REEUSP-2024-0149en (PMC11649066; doi:10.1590/1980-220X-REEUSP-2024-0149en)
Supplement: Translation

# Translation: Atributos da atenção primária à saúde na visão dos profissionais de saúde: revisão de escopo

**DOI:** 10.1590/1980-220X-REEUSP-2024-0149pt

**Authors:** Brenda Lorrana de Almeida Gomes, Renata Sant’ana Braga de Sousa, Renan Felipe Neves Mota, Cynthia Assis de Barros Nunes, Nayara Figueiredo Vieira, Nunila Ferreira de Oliveira, Roxana Isabel Cardozo Gonzalez, Valéria Pagotto

**Affiliations:** 1 Universidade Federal de Goiás, Faculdade de Enfermagem, Goiânia, GO, Brasil.; 2 Universidade Federal de Goiás, Faculdade de Enfermagem, Programa de Pós-graduação em Enfermagem, Goiânia, GO, Brasil.; 3 Universidade Federal de Catalão, Catalão, GO, Brasil.

**Keywords:** Atenção Primária à Saúde, Enfermagem, Acessibilidade aos Serviços de Saúde, Integralidade em Saúde Pesquisa sobre Serviços de Saúde

## Abstract

**Objetivo:**

Identificar as evidências científicas sobre o desempenho da Atenção Primária à Saúde (APS), com base na presença e extensão de seus atributos, na visão dos enfermeiros e médicos.

**Método:**

Revisão de escopo conforme recomendações da *Joanna Brigss Institute*, desenvolvida entre janeiro de 2023 em oito bases de dados e atualizada em dezembro de 2023. Foram utilizadas as palavras-chave “*primary health care assessment*” e “*quality of primary health care*”. Foram incluídos artigos em qualquer língua, que utilizaram o instrumento PCATool-Brasil, versões profissionais, com população de enfermeiros e médicos.

**Resultados:**

Foram incluídos 19 estudos, publicados de 2012 a 2022, em nove estados brasileiros. A média geral do desempenho da APS variou de 6,5 a 8,2 e, do total, somente dois indicaram baixo desempenho dos serviços de APS. Sobre os atributos, a acessibilidade apresentou pior desempenho e orientação familiar o melhor em todos os estudos. Ter especialização e vínculo estatutário foram predominantemente frequentes como fatores associados.

**Conclusão:**

Os resultados mostram que o acesso aos serviços de saúde ainda precisa ser fortalecido e/ou ampliado e que, por outro lado, fatores como a formação e a vinculação do trabalhador fortalecem o desempenho da APS.

## INTRODUÇÃO

A Atenção Primária à Saúde (APS) é considerada a porta de entrada da população brasileira aos serviços de saúde^(1)^ e a ordenadora da Rede de Atenção à Saúde (RAS)^(2)^. Sua consolidação e concretização como uma política de abrangência nacional no Brasil, representa um dos avanços mais relevantes do Sistema Único de Saúde (SUS) nas últimas décadas^(1,2)^.

Devido a sua magnitude e atribuições nas RAS, a APS ampliou-se de 8 mil equipes, em 2007, para mais de 42 mil equipes de saúde da família, em 2017^(3)^, levando à necessidade de avaliação de seu desempenho em diferentes dimensões. Ainda, diante das mudanças epidemiológicas e demográficas da população, bem como de processos de trabalho e da oferta de serviços na APS ao longo do tempo, a avaliação se torna imperativa para acompanhamento da efetividade de suas ações^(3,4)^. As pesquisas avaliativas no Brasil impulsionaram o conhecimento sobre a APS, e elas apontam que uma APS forte leva à maior satisfação de seus usuários e, consequentemente, à melhora nos indicadores de saúde^(4)^.

Uma das propostas para avaliar o desempenho dos serviços de APS se dá por meio de seus elementos estruturantes, definidos como atributos, classificados em essenciais (acesso de primeiro contato, longitudinalidade, integralidade e coordenação do cuidado) e derivados (orientação comunitária e familiar)^(5)^. O *Primary Care Assessment Tool* (PCATool) é o instrumento que mensura a presença e extensão dos atributos da APS, na visão de profissionais (enfermeiros e médicos) e usuários^(6)^. O PCATool baseia-se no modelo de avaliação da qualidade organizado em aspectos de estrutura, processo e resultado dos serviços avaliados^(7)^.

No Brasil, a aplicabilidade do PCATool para avaliação do desempenho da APS foi realizada em diferentes localidades do país com abrangência municipal, regional e microrregional, tanto com usuários quanto com profissionais^(6)^. Em 2017, uma revisão sistemática reuniu 22 estudos com usuários e mostrou que a longitudinalidade foi o atributo com pior desempenho^(8)^.

Na visão de profissionais, algumas pesquisas também foram realizadas utilizando-se o PCATool^(9, 10, 11, 12)^, porém, não foram localizados estudos de revisão reunindo as evidências sobre desempenho da APS na visão dos profissionais de saúde. Este estudo possibilita, portanto, identificar as evidências disponíveis relacionadas à avaliação dos serviços de APS, com base na presença e extensão de seus atributos, ampliando o reconhecimento de fragilidades e potencialidades. Diante disso, o objetivo deste estudo foi identificar as evidências científicas sobre o desempenho da APS, com base na presença e extensão de seus atributos, na visão dos enfermeiros(as) e médicos(as).

## MÉTODO

### Desenho do Estudo

Trata-se de uma revisão de escopo, baseada na metodologia proposta pela *Joanna Briggs Institute* ^(13)^. O estudo foi desenvolvido por meio de cinco etapas: definição da questão de pesquisa; levantamento de dados sobre estudos relevantes; seleção dos estudos; mapeamento dos dados; análise, apresentação e síntese dos resultados. Ainda, utilizou-se as recomendações previstas no *Prisma Extension for Scoping Reviews* (Primas-ScR)^(14)^.

### Pergunta Norteadora

Para elaborar a pergunta norteadora utilizou-se a estratégia mnemônica PPC (*Population, Concept e Context*). Para este estudo, consideramos como população (P) os enfermeiros e médicos da APS; o conceito de interesse (C) foi a avaliação da presença e extensão dos atributos da APS por meio do PCATool-Brasil, e o contexto (C) foi a APS. Assim, a questão central desta revisão de escopo foi: “*Qual é o desempenho dos serviços de APS no Brasil, com base na presença e extensão de seus atributos, na visão dos enfermeiros(as) e médicos(as)?*

### Critérios de Seleção

Nesta revisão, foram considerados estudos observacionais analíticos (transversal e de coorte), que utilizaram o instrumento PCATool-Brasil, versão profissionais. Incluíram-se artigos publicados em qualquer língua, não limitados temporalmente, com o descritor em qualquer campo. Após identificação dos estudos nas bases, foram excluídos: estudos duplicados, protocolos, projetos ou *preview* de pesquisas e estudos cujo objetivo não correspondia à pergunta de pesquisa.

### Coleta de Dados

Foi realizada a seleção bibliográfica com a utilização da base de dados, *Scopus*, Sistema Online de Busca e Análise de Literatura Médica (MEDLINE) (acesso via PubMed), Embase, CINAHL, BDENF E LILACS e SCIELO. A coleta dos artigos foi realizada em janeiro de 2023. Foram utilizadas as palavras-chave: “*primary health care assessment*” OR “*quality of primary health care*”.

Todos os artigos encontrados em cada uma das bases foram baixados no formato de planilha do Excel e agrupados em uma única planilha. Os artigos duplicados foram excluídos utilizando-se a ferramenta de remoção de duplicata do Excel, a partir da coluna de título do artigo. A leitura dos títulos, resumos e textos completos foi realizada por dois revisores, de forma independente e, na ocorrência de discordâncias, as publicações foram analisadas por uma terceira pesquisadora. Os artigos elegíveis foram lidos na íntegra e foram incluídos os que preenchiam os critérios de inclusão.

### Análise de Dados

A extração dos dados foi realizada com base em um formulário desenvolvido pelos autores e baseado no formulário sugerido pelo manual JBI^(13)^. Os dados extraídos incluíram título, autores, ano de publicação, país no qual o estudo foi realizado, tipo de estudo, objetivos do estudo, métodos do estudo, população e tamanho amostral, abrangência do estudo (municipal, estadual ou nacional), resultados gerais (incluindo escore geral do PCATool-Brasil, atributo com menor e com maior escore, e fatores associados investigados por meio de análise de regressão).

Com base nos dados extraídos, fez-se uma análise descritiva das informações coletadas utilizando-se a distribuição de frequência. Ainda, elaborou-se dois quadros: um como os dados das publicações selecionadas e outro sintetizando todos os valores encontrados dos escores dos atributos da APS.

## RESULTADOS

Do total de 1123 artigos identificados, 189 foram selecionados para leitura do resumo. Desses, foram excluídos 4 por duplicidade e 163 que não contemplavam a temática do estudo. Foram elegíveis para leitura 22 artigos, dos quais 14 foram excluídos pelas seguintes razões: incluir gestores no cálculo do escore do PCATool e utilizar usuários como população. Assim, dos 22 artigos selecionados, restaram 8. Ao analisar a lista de referências desses artigos, foram incluídos 11 artigos para essa revisão. Ao final foram incluídos 19 artigos (Figura 1).

**Figura 1:** Fluxograma de busca dos artigos, segundo recomendações PRISMA-ScR, sobre o processo de seleção das publicações da revisão, Brasil, 2023.

O Quadro 1 apresenta a síntese dos artigos incluídos na revisão. Nele foram apresentados os locais de realização dos estudos, objetivos, população, abrangência dos estudos e principais resultados.

**Quadro 1:** Síntese dos artigos incluídos na revisão – Goiânia, GO, Brasil, 2023.

	Autor	País/Estado/Cidade	Objetivo	População	Abrangência do estudo	Resultados gerais
**1**	Costa et al.^(15)^	Brasil Minas Gerais Juiz de Fora	Avaliar a aplicação dos atributos da APS na perspectiva dos profissionais médicos das unidades básicas de saúde	94 médicos	Municipal 63 unidades básicas de saúde (49 urbanas e 14 rurais)	**Escore geral:** 7,05. **Maior escore:** orientação familiar (8,72 DP 1,76) e coordenação/sistema de informações (8,72 DP 1,96). **Menor escore:** acessibilidade (4,02 DP 3,99). **Fatores associados:** não analisados. Correlação positiva entre: (i) maior escore dos atributos longitudinalidade e coordenação/integração do cuidado e maior tempo de atuação na APS e; (ii) maior escore nos atributos longitudinalidade e orientação familiar para aqueles que cursaram pós-graduação (especialização, residência ou mestrado).
**2**	Bomfim et al.^(16)^	Brasil Mato Grosso do Sul 29 municípios	Analisar a qualidade da atenção à saúde do adulto nos serviços de APS na perspectiva de usuários e profissionais	176 enfermeiros 56 médicos	Estadual 29 munícipios representado 4 macrorregiões do estado de Mato Grosso do Sul	**Escore geral:** 7,11 (DP 1,05) **Maior escore:** Orientação familiar 8,57 (DP 1,96) **Menor escore:** Acessibilidade 3,94 (DP 1,31) **Fatores associados:** não analisados. Na comparação dos escores dos profissionais com os dos usuários, houve diferenças significativas em todos os atributos.
**3**	Maia et al.^(12)^	Brasil Goiás 10 municípios	Avaliar a APS com base em seus atributos, sob a perspectiva dos profissionais, verificando fatores que se associam à melhor atenção.	41 enfermeiros 31 médicos	Regional 10 municípios do Estado de Goiás. 36 UBS e 45 equipes de saúde da família dos 10 municípios.	**Escore geral:** 7,26 (DP 0,78) **Maior escore:** Orientação familiar (8,82 DP 1,3) **Menor escore:** Acessibilidade 3,71 (DP 1,26) **Fatores associados:** Ser médico do Programa Mais Médicos (PMM), foi associado aos escores geral (β = 0,942; p = 0,001). essencial (β = 0,948; p = 0,001) e derivado (β = 1,066; p = 0,006).
**4**	Costa et al.^(17)^	Brasil Maranhão São José de Ribamar	Avaliar a qualidade dos serviços de APS no município.	73 enfermeiros e médicos	Municipal 35 UBS 44 equipes	**Escore geral:** 7,54 (DP não apresentado) **Maior escore:** coordenação/ sistema de informação (8,93 DP 0,4). **Menor escore:** acessibilidade (3,91 DP 0,4). **Fatores associados:** não analisados. Na comparação dos escores dos profissionais com os dos usuários, houve diferenças significativas em todos os atributos.
**5**	Silva e Alves^(18)^	Brasil Minas Gerais Diamantina	Avaliar o grau de implantação dos atributos da APS como indicador da qualidade da assistência prestada às crianças.	8 enfermeiros 14 médicos	Municipal 14 equipes (8 urbanas, 4 rurais e 2 mistas)	**Escore geral:** 6,9 (DP 1,1) **Maior escore:** integralidade, sistemas de informações (9,0) e orientação familiar (8,9). **Menor escore:** acessibilidade (4,2). **Fatores associados:** não analisados. Teste de comparação entre o desempenho dos serviços na área urbana e rural, mostrou que acessibilidade apresentou maior escore entre profissionais da zona rural (5,3), do que os da zona urbana (3,7) (p = 0,042)
**6**	Vieira et al.^(19)^	Brasil Minas Gerais Lagoa Santa	Comparar a presença e a extensão dos atributos da APS no geral e no desempenho das ACH.	17 enfermeiros 11 médicos	Municipal Lagoa Santa 17 equipes de saúde da família	**Escore geral:** 6,8 (DP 0,9) **Maior escore:** orientação familiar (8,6 DP 1,3) **Menor escore:** acessibilidade (3,8 DP 1,0). **Fatores associados:** não analisados.
**7**	Gomes e Fracolli^(20)^	Brasil São Paulo Municípios da região de Presidente Prudente	Avaliar se os atributos essenciais e derivados da Atenção Primária à Saúde estão presentes na Estratégia Saúde da Família.	44 enfermeiros 39 médicos	Regional 21 municípios da Rede Regional de Atenção à Saúde de Presidente Prudente	**Escore geral:** 7,88 (DP 0,90) **Maior escore:** Coordenação-sistema de informação 8,74 (1,42) **Menor escore:** Acessibilidade: 5,57 (1,42) **Fatores associados:** não analisados. Apresenta somente os escores dos atributos acessibilidade, longitudinalidade e coordenação- integração de cuidados.
**8**	Piovesan et al.^(21)^	Brasil Rio Grande do Sul	Avaliar, na experiência dos profissionais, a qualidade da atenção primária à saúde de municípios de procedência de crianças e adolescentes com HIV, acompanhados em serviço especializado.	167 enfermeiros 245 médicos	Estadual 25 municípios do RS	**Escore geral:** 6,64 (DP 1,02) **Maior escore:** coordenação, sistema de informação (8,19 (1,87 em UBS e 8,24 (1,50) em ESF). **Menor escore:** acesso de primeiro contato (3,89 DP 11,78 em UBS e 3,80 (1,09) em ESF **Fatores associados:** Sexo feminino(p = 0,009); formação profissional clínico-geral (p < 0,001); o vínculo no serviço estatutário (0,029); o cargo de trabalho do profissional como coordenador do serviço(p = 0,087) foram associados ao melhor desempenho da APS.
**9**	Santos et al.^(11)^	Brasil Município no sudoeste de Goiás	Avaliar a presença e a extensão dos atributos da APS por profissionais da saúde que atuam na APS de município do sudoeste goiano.	17 enfermeiros 13 médicos	Municipal 16 Unidades básicas de saúde de um município do sudoeste goiano	**Escore geral:** 7,2 (não apresenta DP) **Maior escore:** Orientação familiar 8,7 **Menor escore:** Acessibilidade: 3,4 **Fatores associados:** tempo de atuação ≤ 5 anos e Tempo de atuação ≥ 6 anos; profissionais de saúde com e sem especialização na área de atuação
**10**	Sousa et al.^(22)^	Brasil Pará Canaã dos Carajás	Avaliar a presença e a extensão dos atributos essenciais e derivados da atenção primária em saúde no programa de controle da hanseníase, sob a ótica do enfermeiro.	11 enfermeiros	Municipal 8 equipes ESF 3 UBS 6 USF	**Escore geral:** 8,2 (±0,7) **Maior escore:** Integralidade- serviços disponíveis 9,5 (±0,4) **Menor escore:** Acesso 5,9 (±1,4) **Fatores associados:** não analisados. Somente análise descritiva.
**11**	Oliveira et al.^(23)^	Brasil Goiás Região noroeste de Goiânia	Analisar perfil de formação e qualificação dos profissionais da Estratégia Saúde da Família (ESF) e os fatores associados com a qualidade dos serviços de APS da região noroeste de Goiânia	44 enfermeiros 48 médicos	Municipal 18 Unidades básicas de saúde da região noroeste de Goiânia	**Escore geral:** 6,7 (1,0) **Maior escore:** Integralidade- serviços prestados: 8,0 (1,4) **Menor escore:** Acessibilidade: 4,6 (1,0) **Fatores associados:** Analisados, porém após análise ajustada, ter especialização, realização de capacitação e aperfeiçoamento profissional não mantiveram associação com o alto escore.
**12**	Ferreira et al.^(10)^	Brasil Minas Gerais Passos	Avaliar, utilizando o instrumento PCATool, a extensão dos atributos essenciais e derivados da APS na perspectiva dos enfermeiros das USF e UBS convencionais do Município de Passos, MG.	27 enfermeiros	Municipal 19 USF 9 UBS	**Escore geral:** Para UBS 6,8 (1,16), para ESF 7,4 (0,64) **Maior escore:** Orientação familiar UBS 8,61 (1,42), ESF 9,18 (1,16) **Menor escore:** Acessibilidade: UBS 4,44 (0,97), ESF 4,21 (0,86) **Fatores associados:** não analisados. Somente análise descritiva.
**13**	Nascimento et al.^(24)^	Brasil Rio Grande do Sul Santa Maria	Avaliar a qualidade da atenção à saúde da criança e adolescente vivendo com HIV, entre os diferentes tipos de serviço de APS em Santa Maria (RS) por meio do PCATool-Brasil, versão Profissionais.	34 enfermeiros 60 médicos 24 cirurgiões dentistas	Municipal 31 serviços de APS 18 UBS 13 ESF	**Escore geral:** 6,49 (1,15) **Maior escore:** coordenação, sistema de informação 8,44 (1,61) **Menor escore:** acesso 4,02 (1,30) **Fatores associados:** Foram associados ao alto escore (>6,6): possuir outro emprego, atuar em na ESF e ter vínculo estatutário.
**14**	Turci et al.^(25)^ ^¥^	Brasil Minas Gerais Belo Horizonte	Avaliar o desempenho da APS em Belo Horizonte, Minas Gerais, Brasil, utilizando o questionário PCATool entre enfermeiros das equipes de saúde da família e gerentes	463 enfermeiros *138 gestores	Municipal 538 equipes 147 UBS	**Escore geral:** 0,75 (IC 0,74–0,76). **Maior escore:** Acesso, primeiro contato (0,95 IC 0,94–0,97) **Menor escore:** acessibilidade (0,45 IC 0,43–0,46) **Fatores associados:** Disponibilidade de equipamentos e outros insumos (RP ajustada = 1,57). Formação dos profissionais em saúde da família (RP = 1,44). Presença do médico por mais de 30 horas semanais (RP = 1,42). 4 ou mais equipes por unidade básica de saúde (RP = 1,09).
**15**	Silva et al.^(26)^	Brasil Minas Gerais Microrregião de saúde Alfenas	Avaliar os atributos da APS triangulando e comparando o ponto de vista de atores sociais implicados no processo assistencial.	19 enfermeiros 15 médicos	Regional 11 municípios de uma microrregião do Estado de MG 33 unidades da ESF	**Escore geral:** 7,40 (DP 0,77) **Maior escore:** Coordenação, sistemas de Informação (8,95 DP 1,33) **Menor escore:** Acesso de Primeiro Contato, Acessibilidade (4,13 DP1,27) **Fatores associados:** não analisados. Os atributos foram comparados entre os grupos adulto, cuidadores de crianças de 0 a 2 anos e profissionais, mostrando concordância na avaliação baixa para o Acesso de Primeiro Contato, Acessibilidade e alta para a Longitudinalidade.
**16**	Araújo et al.^(27)^	Brasil Distrito Federal	Avaliar a presença e a extensão dos atributos no cuidado integral à saúde da criança, comparando as UBT que atuam com o modelo tradicional com as UBSF do DF, na percepção de usuários e profissionais.	16 profissionais (oito por modelo de UBS)	Regional Duas regiões do DF 15 UBT 19 ESF	**Escore geral:** UBT 7,01 (0,54); ESF 6,62 (0,61) **Maior escore:** UBT coordenação SI 9,19 (0,88); ESF orientação familiar 8,55 (1,64) **Menor escore:** Acessibilidade: UBT 3,21 (0,96); ESF 2,75 (0,65) **Fatores associados:** não analisados.
**17**	Chomatas et al.^(28)^	Brasil Paraná Curitiba	Avaliar a presença e a extensão dos atributos da atenção primária à saúde no município de Curitiba, comparando unidades tradicionais com unidades da Estratégia Saúde da Família, por meio da experiência de seus profissionais de saúde.	99 enfermeiros 91 médicos	Municipal 45 unidades com ESF 47 UTRAD	**Escore geral:** UTRAD 6,7 (6,6–6,8); ESF 7,4 (7,3–7,5) **Maior escore:** UTRAD Coordenação SI 8,3 (8,1–8,5); ESF Coordenação SI 8,5 (8,2–8,7) e Orientação familiar 8,5 (8,3–8,7) **Menor escore:** UTRAD Acessibilidade 4,1 (3,9–4,2); ESF acessibilidade 4,2 (4,0–4,4) **Fatores associados:** unidades com ESF (RP = 1,44) estavam associadas com Alto Escore Geral da APS. Ser profissional médico em relação ao enfermeiro (RP = 0,87) estava associado com uma diminuição da prevalência de Alto Escore Geral da APS. Nos modelos multivariáveis estratificados, entre os médicos, a presença de alto escore geral da APS estava associada positivamente com unidade com ESF e número de consultas semanais (10 consultas semanais). Para os enfermeiros, possuir formação em enfermagem comunitária estava associado positivamente com a presença de alto escore geral da APS
**18**	Vitória et al.^(29)^	Brasil Santa Catarina Chapecó	Avaliar a adequabilidade dos elementos da estrutura e dos processos da APS em Chapecó.	47 enfermeiros 51 médicos	Municipal 24 UBS	**Escore geral:** 7,09 (6,95–7,24) **Maior escore:** Coordenação- sistema de informação: 8,8 (8,55–9,07) **Menor escore:** Acesso 3,6 (3,40–3,90) **Fatores associados:** Não analisados. Discute sobre formação e processo de trabalho, mas não os relaciona com os escores.
**19**	Castro et al.^(9)^	Brasil Rio Grande do Sul Porto Alegre	Comparar a qualidade da atenção à saúde do adulto entre os diferentes tipos de serviços de APS de Porto Alegre, Rio Grande do Sul,	114 enfermeiros 226 médicos	Municipal 26 UBS 31 ESF	**Escore geral:** UBS 6,58, ESF 7,08 **Maior escore:** Em ambos Coordenação SI 9,00 e 9,02 **Menor escore:** Ambos Acesso 3,14 e 3,53 **Fatores associados:** Especialidade na área de APS e oferta de educação continuada pelo serviço associaram-se com alto escore geral.

O período das publicações variou de 2012 a 2022, sendo 65% nos últimos 6 anos. Quanto aos locais, os estudos foram desenvolvidos em 9 estados brasileiros, com maior concentração em Minas Gerais (6 estudos), seguido de Goiás (3 estudos), Rio Grande do Sul (3 estudos), Maranhão, Mato Grosso do Sul, Pará, São Paulo, Paraná, Santa Catarina e Distrito Federal (1 estudo em cada). Em 68,4% dos estudos, a abrangência foi municipal e nos demais houve abrangência regional e/ou estadual (Quadro 1).

Quanto à população, um estudo incluiu somente médicos, três estudos focalizaram somente enfermeiros, onze pesquisas incluíram enfermeiros e médicos, e quatro delas se voltavam para enfermeiros, médicos e cirurgiões-dentistas. Ainda, dois compararam a visão de profissionais com a de usuários^(16,17)^, e um com usuários cuidadores^(18,26)^. Dois estudos ainda compararam a visão dos gestores com a dos profissionais^(20,25)^. Esses estudos foram incluídos por apresentar o escore do PCATool na visão de profissionais, separado dos outros grupos (usuários, cuidadores e gestores), os quais não foram considerados na análise desta revisão (Quadro 1).

Destaca-se que na descrição dos objetivos, em oito estudos, os autores utilizam os termos avaliação da presença e extensão dos atributos da APS, em seis, avaliação da qualidade dos serviços de APS, um menciona desempenho da APS, um, grau de implantação dos atributos e nos demais outras descrições similares às duas primeiras (Quadro 1).

Sobre os fatores associados apresentados, em seis estudos foram realizadas análises estatísticas que permitem inferir associação de variáveis independentes com o escore do PCATool^(11,12,24,25,28)^. A variável ter especialização na área de atuação foi associada ao maior desempenho dos serviços de APS em quatro estudos^(9,11,25,28)^. O vínculo profissional estatutário foi associado ao maior escore da APS em dois estudos^(21,24)^. Atuar em unidades básicas de saúde com estratégia de saúde da família foi associada em dois estudos^(25,28)^ (Quadro 1).

Algumas variáveis relacionadas ao profissional médico tais como ser médico do Programa Mais Médicos (PMM)^(12)^; formação profissional clínico-geral^(21)^; presença do médico por mais de 30 horas semanais^(25)^ foram associadas.Outras variáveis foram associadas, porém cada uma em um estudo: sexo feminino^(21)^, ser coordenador do serviço^(21)^, tempo de atuação ≤ 5 anos^(11)^, possuir outro emprego^(24)^, disponibilidade de equipamentos e outros insumos^(25)^, ser profissional médico em relação ao enfermeiro^(28)^, oferta de educação continuada pelo serviço^(9)^.

A Tabela 1 apresenta os escores dos atributos, com heterogeneidade na apresentação dos valores. Em todos os estudos foi apresentado o escore geral do PCATool Brasil, o qual variou de 6,49 a 8,20. Dos 19 estudos, somente dois apresentaram escore abaixo do ponto de corte do instrumento (6,6)^(9,24)^.

**Tabela 1:** Escore dos atributos da Atenção Primária à Saúde – Goiânia, GO, Brasil, 2023.

	Estudos	Atributos essenciais média (DP)				Atributos derivados média (DP)		Escore geral média (DP)		
		Acessibilidade	Longitudina-lidade	Coordenação	Integralidade	Orientação familiar	Orientação comunitária	Essenciais	Derivados	Geral
**1**	Costa et al.^(15)^	4,02 (3,99)	7,12 (2,62)	7,22 (3,08)^CI^ 8,72 (1,96)^SI^	6,74 (3,77)^SD^ 8,31 (4,25)^SP^	8,72 (1,76)	7,57 (3,00)	6,93 (3,47)	7,95 (2,70)	7,05 (3,40)
**2**	Bomfim et al.^(16)^	3,94 (1,31)	7,7 (1,42)	7,49 (1,78)^CI^ 8,46 (1,71)^SI^	7,1 (1,49)^SD^ 6,63 (2,26)^SP^	8,57 (1,96)	7,03 (2,09)	–	–	7,11 (1,05)
**3**	Maia et al.^(12)^	3,71 (1,26)	7,29 (1,29)	7,24 (1,48)^CI^ 8,48 (1,45)	7,32 (1,02) 8,04 (1,35)	8,82 (1,31)	7,19 (1,69)	7,01 (0,75)	8,00 (1,16)	7,26 (0,78)
**4**	Costa et al.^(17)^	3,91 (0,4)	7,57 (*)	8,15 (*)^CI^ 8,93 (0,4)	7,51 (*) 8,19 (*)	8,90 (0,4)	7,58 (*)	7,28 (0,2)	8,02 (0,3)	7,54 (*)
**5**	Silva e Alves^(18)^ urbana	3,7 (0,3)**	7,1 (0,5)	6,7 (0,5)^CI^ 8,8 (0,4)^SI^	5,9 (0,6)^SD^ 7,6 (0,5)^SP^	9,2 (0,3)	5,8 (0,4)	6,6 (0,4)	–	6,9 (0,3)
	Silva e Alves^(18)^ rural	5,3 (0,6)**	5,6 (0,7)	6,2 (0,4)^CI^ 9,3 (0,4)^SI^	5,6 (0,6)^SD^ 7,0 (0,6)^SP^	8,0 (0,8)	5,7 (0,8)	6,5 (0,4)	–	6,6 (0,4)
	Silva e Alves^(18)^ Geral	4,2 (1,4)	6,59 (1,76)	8,99 (1,18) 6,03 (1,87)	6,03 (1,87) 7,46 (1,68)	8,94 (1,26)	5,79 (1,74)	6,7 (1,2)	6,86 (1,10)	6,9 (1,1)
**6**	Vieira et al.^(19)^	3,8 (1,0)^A^	6,9 (1,1)	7,8 (1,0)^CI^	7,6 (1,1)^SD^ 6,6 (1,6)^SP^	8,6 (1,6)	6,5 (1,4)	6,5 (0,8)	7,6 (1,2)	6,8 (0,9)
**7**	Gomes e Fracolli^(20)^	5,57 (1,42)	7,71 (1,26)	7,70 (1,23)^CI^ 8,74 (1,42)^SI^	8,29 (1,24)^SD^ 8,40 (1,57)^SP^	8,53 (1,74)	8,12 (1,41)	7,73 (0,85)	–	7,88 (0,90)
**8**	Piovesan et al.^(21)^ UBS	3,96 (1,78)	6,74 (1,34)	7,03 (1,51)^CI^ 8,19 (1,87)^SI^	6,49 (1,77)^SD^ 6,37 (2,77)^SP^	–	–	6,47 (1,10)	–	–
	Piovesan et al.^(21)^ ESF	3,80 (1,09)	7,17 (1,24)	6,87 (1,50)^CI^ 8,24 (1,50)^SI^	7,17 (1,52)^SD^ 7,66 (1,96)^SP^	–	–	6,82 (0,88)	–	–
**9**	Santos et al.^(11)^ ***	3,4	7,0	7,1^CI^ 8,4^SI^	7,3 ^SD^ 8,2 ^SP^	8,7	7,2	–	–	7,2
**10**	Sousa et al.^(22)^	5,9 (1,4)	8,1 (1,6)	7,8 (1,9)	9,5 (0,4)^SD^ 8,8 (1,0)^SP^	7,8 (2,8)	7,8 (1,8)	8,1 (0,6)	7,9 (1,6)	8,2 (0,7)
**11**	Oliveira et al.^(23)^	4,6 (1,0)	6,8 (1,2)	6,9 (1,3)^CI^ 6,5 (1,4)^SI^	6,5 (1,4)^SD^ 8,0 (1,4)^SP^	7,6 (1,6)	6,5 (1,4)	6,5 (0,9)	6,9 (1,4)	6,7 (1,0)
**12**	Ferreira et al.^(10)^ UBS	4,44 (0,97)	7,37 (1,20)	7,57 (0,84)^CI^ 7,36 (2,65)^SI^	5,42 (0,96)^SD^ 7,69 (1,69)^SP^	8,61 (1,42)	5,83 (2,94)	6,65 (1,06)	–	6,80 (1,16)
	Ferreira et al.^(10)^ ESF	4,21 (0,86)	7,88 (1,17)	7,66 (1,18)^CI^ 8,71 (1,82)^SI^	6,13 (0,54)^SD^ 7,92 (1,18)^SP^	9,18 (1,16)	7,43 (1,34)	7,09 (0,68)	–	7,40 (0,64)
**13**	Nascimento et al.^(24)^	4,02 (1,30)	6,70 (1,37)	6,13 (1,54)^CI^ 6,45 (3,04)	6,97 (1,56) 8,44 (1,61)	7,80 (2,36)	5,41 (2,03)	6,45 (1,06)	6,61 (1,88)	6,49 (1,15)
**14**	Turci et al.^(25)^ ^¥^	0,45 (0,43–0,46)	0,83 (0,81–0,85)	0,78 (0,77–0,79)	0,83 (0,82–0,84)	0,68 (0,66–0,71)	0,56 (0,54–0,59)	–	–	0,75 (0,74–0,76)
**15**	Silva et al.^(26)^	4,13 (1,27)	7,86 (1,12)	6,91 (1,47) 8,95 (1,33)	6,80 (1,24) 8,01 (1,25)	8,82 (1,40)	7,68 (1,46)	5,92 (1,20)	–	7,40 (0,77)
**16**	Araújo et al.^(27)^ UBT	3,21 (0,96)	7,53 (1,24)	7,61 (1,60)^CI^ 9,19 (0,88)^SI^	5,97 (0,97)^SD^ 8,36 (1,70)^SP^	7,57 (2,10)	6,67 (2,39)	6,98 (0,64)	7,11 (0,21)	7,01 (0,54)
	Araújo et al.^(27)^ ESF	2,75(0,65)	7,31(1,42)	6,69 (1,83)^CI^ 8,37 (1,36)^SI^	5,31 (1,10)^SD^ 8,35 (1,53)^SP^	8,55 (1,64)	5,61 (1,57)	6,46 (0,65)	7,08 (0,64)	6,62 (0,61)
**17**	Chomatas et al.^(28)^ UTRAD	4,1 (3,9–4,2)	6,0 (5,8–6,1)	6,9 (6,7–7,0)^CI^ 8,3 (8,1–8,5)^SI^	7,1 (7,0–7,2)^SD^ 6,0 (5,7–6,2)^SP^	7,8 (7,6–8,0)	7,6 (7,4–7,8)	6,4 (6,3–6,5)	7,7 (7,5–7,9)	6,7 (6,6–6,8)
	Chomatas et al.^(28)^ ESF	4,2 (4,0–4,4)	6,6 (6,4–6,7)	7,0 (6,8–7,1)^CI^ 8,5 (8,2–8,7)^SI^	7,9 (7,8–8,0)^SD^ 8,3 (8,2–8,5)^SP^	8,5 (8,3–8,7)	8,1 (7,9–8,3)	7,1 (7,0–7,2)	8,3 (8,2–8,5)	7,4 (7,3–7,5)
**18**	Vitória et al.^(29)^	3,6 (3,40–3,90)	6,0 (5,83–6,34)	7,2 (6,97–7,53)^CI^ 8,8 (8,55–9,07)^SI^	7,7 (7,57–7,96)^SD^ 7,6 (7,33–7,89)^SP^	8,6 (8,32–8,91)	6,9 (6,65–7,26)	6,8 (6,71–7,01)	–	7,09 (6,95–7,24)
**19**	Castro et al.^(9)^ UBS	3,14	6,21	6,92^CI^ 9,00^SI^	6,29^SD^ 7,02^SP^	8,21	5,58	6,45	–	6,58
	Castro et al.^(9)^ ESF	3,53	6,53	7,17^CI^ 9,02^SI^	6,50^SD^ 8,27^SP^	8,87	6,75	6,84	–	7,08

*Estudo de Costa et al.^(17)^: valores de Desvio Padrão (DP) ausentes para esses atributos;

^§^Estudo de Vieira et al.^(19)^: informações ausentes por *missing* nos atributos.

^¥^Estudo de Turci et al.^(25)^: escores por regressão;

**Estudo de Silva e Alves^(18)^: valor de Erro Padrão (EP);

***Estudo de Santos et al.^(11)^: não apresenta valores de Desvio Padrão (DP);

Em relação aos atributos essenciais, em 16 estudos (84,2%) foi apresentado o escore geral essencial variando de 5,9 a 8,1; enquanto, o escore geral dos atributos derivados foi apresentado em 10 estudos (52,6%), variando de 4,3 a 8,3. Destaca-se que o escore geral essencial foi menor que os derivados em todos os estudos, com exceção de dois estudos^(19,22)^.

Entre os quatro atributos essenciais (acessibilidade, longitudinalidade, integralidade e coordenação de cuidado), os menores escores foram observados para acessibilidade cujos valores foram abaixo do ponto de corte em todos os estudos. O escore da longitudinalidade variou de 5,6 a 8,1, sendo que em 6 estudos (31,6%) foi abaixo de 6,6. Já os atributos de coordenação do cuidado e integralidade, apresentaram escores mais elevados que os dois primeiros, com valores acima de 8,5 em 17 (89,4%) e 11 (58,0%) estudos respectivamente. Quanto aos dois atributos derivados (orientação familiar e comunitária), em todos os estudos os escores observados foram acima de 7,5 para a orientação familiar. Já a orientação comunitária, em 8 estudos (42,1%), o escore foi abaixo de 6,6. Já os atributos de coordenação do cuidado e integralidade, apresentaram escores mais elevados que os dois primeiros. No caso do atributo coordenação do cuidado, 11 (57,9) artigos apresentaram valores acima de 8,5. Quanto aos dois atributos derivados (orientação familiar e comunitária), na maior parte dos estudos foram observados escores superiores a 7,5 para a orientação familiar. Já na orientação comunitária, em 10 estudos (52,6%), o escore foi abaixo de 6,6.

Em relação aos estudos que apresentaram análises distintas de acordo com a tipologia da unidade de saúde, os que fizeram comparação entre os escores dos atributos na ESF e UBS, apontaram que na ESF os escores foram superiores em grande parte dos atributos^(8,12,19)^. O estudo que realizou a análise distinta entre USF rurais e urbanas demonstrou que o desempenho da USF urbana foi superior à rural^(5)^.

## DISCUSSÃO

Os resultados dessa revisão de escopo apontam que o PCATool tem sido utilizado como ferramenta de avaliação da presença e extensão dos atributos da APS, em diferentes contextos nacionais. Embora o escore geral dos atributos seja homogêneo entre as diferentes localidades, os valores médios de cada um dos atributos são heterogêneos entre si, indicando que o desempenho dos serviços de APS no Brasil pode estar atrelado a forma de organização e envolvimento da gestão no processo de trabalho na APS, evidenciando que apesar das recomendações das políticas há aspectos específicos que precisam ser aprimorados em cada um dos atributos conforme a realidade de cada serviço.

Apesar disso, nos estudos incluídos, a maior proporção apresentou alto desempenho nos atributos essenciais e derivados (acima de 6,6). A Estratégia Saúde da Família é um dos programas estruturantes do Sistema Único de Saúde (SUS) e sua expansão contribuiu para mudanças no perfil epidemiológico da população nas últimas três décadas^(30)^. Estudos analisando indicadores de saúde nesse período, que corresponde à grande parte da existência do SUS, mostram queda da mortalidade por doenças transmissíveis, da morbimortalidade materno-infantil e aumento da expectativa de vida saudável da população^(31,32)^, além de menor risco de morte em pessoas que utilizam a APS^(33)^. Os resultados da presente pesquisa, somados às evidências de pesquisas que mostram mudanças significativas de indicadores de saúde, reafirmam o papel da APS como porta de entrada ao SUS, como ordenadora do cuidado e como centro de comunicação com toda a Rede de Atenção, indicando a sustentabilidade e consolidação desse modelo de atenção à saúde adotado no Brasil^(34,35)^.

A maioria dos estudos dessa revisão mostraram que os profissionais avaliaram os atributos essenciais da APS como fortemente orientados. No entanto, a perspectiva dos usuários, numa revisão sistemática com 22 artigos entre os anos de 2007 e 2015, mostrou melhor avaliação no atributo “acesso de primeiro contato”, enquanto a pior avaliação se deu na dimensão acessibilidade, integralidade, orientação familiar e orientação comunitária^(8)^. A visão de profissionais sobre os atributos da APS apresenta uma perspectiva diferente quando comparada à dos usuários e, embora sejam ambos substanciais para compreensão do desempenho dos serviços de APS, neste artigo, o enfoque será na avaliação da perspectiva de profissionais de saúde.

O baixo desempenho do atributo acessibilidade, na visão de profissionais, foi evidenciado em todos os estudos incluídos. O acesso pode ser diferenciado em duas grandes dimensões, geográfica e sócio-organizacional. Enquanto a primeira engloba os fatores que podem aumentar a dificuldade de alcançar o serviço de saúde – tais como distância, tempo e custo para o deslocamento – a segunda se refere a todas as demais características que envolvem o acesso – tais como a maneira de ingressar e receber o cuidado na unidade de saúde^(7)^. No PCATool, as nove questões que compõem a segunda dimensão, contemplam itens como horário de funcionamento aos finais de semana, noturno, aconselhamento por ferramenta de comunicação virtual (como aplicativos de mensagens)^(6)^, as quais além de apresentar divergências entre os diversos cenários brasileiros, pode não ser a realidade da política de saúde local. Dados do terceiro ciclo do Programa de Melhoria da Qualidade da Atenção Básica (PMAQ), 2017-2018, em seu componente organizacional, mostrou predomínio de funcionamento das unidades de saúde de segunda a sexta-feira, com pouca oferta em horários alternativos (1,6% delas funcionavam aos sábados e 0,7% aos domingos)^(36)^. Destaca-se que, na avaliação dos usuários, o componente acessibilidade, também apresentou baixo desempenho em revisão sistemática^(8)^, e as questões são similares à versão profissionais. Em geral, as dificuldades que impedem o acesso aos serviços de saúde podem se manifestar de maneira sutil, evidente ou oculta, as quais quando identificadas não recebem os encaminhamentos efetivos para sua solução. Assim, destaca-se a necessidade de aprimoramento dos processos para garantir a entrada e cuidado de qualidade das pessoas na APS, tendo em vista o reconhecido papel desse atributo para redução da morbimortalidade.

Entre os atributos essenciais, a coordenação do cuidado apresentou o maior escore entre os estudos analisados. A coordenação refere-se à articulação entre os diversos níveis de atenção que se estabelece por meio de linhas de comunicação que permitem o fluxo de informações entre os profissionais nos diversos níveis de atenção. Com esse atributo desenvolvido, é possível um acompanhamento contínuo da saúde de cada paciente^(5)^. Assim sendo, reflete-se a capacidade de resolutividade e de funcionamento da RAS, bem como a capacidade estrutural das políticas para uma APS forte. A avaliação satisfatória, evidenciada nesse atributo, talvez possa ser atribuída à percepção da necessidade de envolver a rede de atenção primária e especializada à saúde da população do território. No entanto, destaca-se que a ordenação a partir da APS nem sempre se contribui como barreira, mas, o retorno do usuário para o serviço de APS, conhecido como contrarreferência, é um aspecto que dificulta a continuidade do cuidado, que nem sempre possui integração ou comunicação, o que pode ser aprofundado em pesquisas específicas sobre a coordenação do cuidado.

O atributo orientação familiar apresentou alto desempenho em todos os estudos incluídos nesta revisão. Envolve a interação profissional-usuário-família na tomada de decisões sobre cuidados e tratamento com a saúde, na identificação de problemas na família, além de avaliar a disposição do profissional para abordar assuntos que sejam importantes para a saúde^(6)^. A orientação familiar se efetiva quando o alcance da integralidade fornece uma base para a consideração do indivíduo dentro de seus ambientes; a avaliação das necessidades para a atenção integral considera o contexto familiar e sua exposição a ameaças à saúde; o desafio da coordenação da atenção se defronta com recursos familiares limitados, necessitando de ações para adequação ao contexto familiar. Nessa perspectiva, destaca-se novamente os pressupostos da ESF, de reorganização da prática da atenção à saúde, por meio de modelo de atenção centrado na família com acompanhamento longitudinal. O alto desempenho apontado em todos os estudos pelos profissionais de saúde pode ser reflexo da formação profissional que orienta para esse modelo assistencial, com ênfase na abordagem ampliada da pessoa e de sua família.

No entanto, é importante também avaliar a perspectiva de usuários sobre a orientação familiar, uma vez que, para uma APS forte e resolutiva, é importante que tanto profissionais como usuários e família estejam satisfeitos e engajados com o cuidado, o que, segundo pesquisas sobre essa temática com usuários ^(18,37)^, ainda precisa ser melhor trabalhado e aperfeiçoado. Ainda, é importante destacar a mudança da inclusão de diferentes atribuições aos profissionais de APS previstas na PNAB 2017, as quais, ao descentralizar ações previstas para outros pontos da RAS, pode gerar sobrecarga aos serviços e, consequentemente, levar ao atendimento individualizado em detrimento daquele centrado na pessoa e sua família. Assim, como já apontado em pesquisa anterior, é importante a aproximação dos profissionais do contexto familiar e social e da inclusão da família para o planejamento das intervenções, e também a relevância deste tema para a formação e ainda na promoção da educação permanente em saúde^(18)^.

Entre as variáveis que foram avaliadas como fatores associados nos 19 estudos, ter especialização e vínculo estatutário foram as mais frequentes e serão discutidas por seu potencial de impacto na APS.

Ter especialização ocorreu em quatro estudos ^(9,11,25,28)^. Um estudo realizado com 20 profissionais que cursaram especialização na área de Educação voltado para o SUS, indicou que a formação qualificou o processo de trabalho e agregou maior dinamismo para atender às demandas coletivas e individuais, além de propiciar mudanças no perfil gerencial destacando o trabalho em equipe e propostas interdisciplinares^(38)^. O direcionamento da formação generalista proposta pelas DCN, com vistas a fortalecer a atuação no âmbito do SUS pode seguir para um sentido dicotômico entre habilidades generalistas x especialidades, em detrimento da possibilidade de se direcionar uma formação de carreira voltada para APS, que abarque as necessidades e complexidades inerentes a esse contexto de trabalho^(39)^. A formação generalista não impacta diretamente na escolha dos profissionais médicos para seguir essa carreira ou permanecer na Atenção Primária, esse percurso tende a ser temporário e colabora para uma formação voltada para desenvolvimento de aspectos sociais e de contribuição com a sociedade no entanto, esses profissionais tendem buscar formação especializada, geralmente por justificativa de maior reconhecimento profissional^(40)^. Esses resultados mostram a importância da formação generalista, de qualidade, com aproximação à realidade dos serviços de saúde e ao fortalecimento do SUS, mas também mostram a necessidade de investimentos em formações específicas ao longo da carreira, inclusive especialização(ões) voltadas para APS com vistas a melhorar o desempenho das equipes.

A variável vínculo empregatício estatutário ocorreu em dois estudos^(21,24)^, resultado positivo para a APS, uma vez que a estabilidade profissional efetiva o atributo da longitudinalidade e promove o fortalecimento do vínculo entre o profissional e o usuário^(41)^. Entretanto, cabe mencionar que há evidência de que o contrato administrativo contratado também aparece como associado ao alto desempenho da APS^(42)^. Assim, cabe destacar que a rotatividade profissional constitui um impasse para a expansão e consolidação da APS.

### Limitações do Estudo

Uma limitação do estudo refere-se aos procedimentos para avaliação das evidências encontradas. Porém, a totalidade dos estudos era transversal, com delineamento apropriado para utilização do instrumento PCATool, foco desta revisão. Além disso, é possível que alguns estudos relevantes não tenham sido contemplados pelas limitações das palavras-chave, no entanto acreditamos que quaisquer estudos perdidos não alteram substancialmente o padrão das descobertas.

## CONCLUSÃO

O presente estudo possibilitou sintetizar o conhecimento acerca da presença e extensão dos atributos da APS na visão de profissionais de saúde, enfermeiros e médicos. Os resultados são importantes para o reconhecimento do cenário dos atributos essenciais e derivados nas diferentes regiões do país, o que pode auxiliar enfermeiros e médicos que atuam na assistência e gestão do serviço de APS a refletir para reorganização do processo de trabalho, a partir de seus elementos estruturantes que são os atributos.

Assim, atributos a exemplo do acesso de primeiro contato, que apresentou um baixo desempenho em grande parte dos estudos, podem ser um ponto de partida para identificação de problemas-chave para reorganização de ações nos serviços de APS, as quais serão propulsoras para desenvolvimento de ações que promovam a longitudinalidade, a integralidade e a coordenação do cuidado. Sugere-se que sejam realizados mais estudos primários sobre a qualidade do cuidado na APS, utilizando outras metodologias avaliativas, tanto de natureza quantitativa quanto qualitativa. Assim, fenômenos que não foram contemplados pelo instrumento PCATool poderão ser identificados.
